# The population genetic structure of *Biomphalaria choanomphala* in Lake Victoria, East Africa: implications for schistosomiasis transmission

**DOI:** 10.1186/s13071-014-0524-4

**Published:** 2014-11-19

**Authors:** Claire J Standley, Sara L Goodacre, Christopher M Wade, J Russell Stothard

**Affiliations:** School of Life Sciences, University of Nottingham, Nottingham, NG7 2RD UK; Department of Zoology, Wolfson Wellcome Biomedical Laboratory, Natural History Museum, Cromwell Road, London, SW7 5BD UK; Department of Parasitology, Liverpool School of Tropical Medicine, Pembroke Place, Liverpool, L3 5QA UK; Present address: Milken Institute School of Public Health, George Washington University, Washington, D.C. 20052 USA

**Keywords:** *Biomphalaria choanomphala*, *Schistosoma mansoni*, Population structure, Population genetics

## Abstract

**Background:**

The freshwater snail *Biomphalaria* acts as the intermediate host of *Schistosoma mansoni*, a globally important human parasite. Understanding the population structure of intermediate host species can elucidate transmission dynamics and assist in developing appropriate control methods.

**Methods:**

We examined levels of population genetic structure and diversity in 29 populations of *Biomphalaria choanomphala* collected around the shoreline of Lake Victoria in Uganda, Kenya and Tanzania, where *S. mansoni* is hyper-endemic. Molecular markers were utilized to estimate the degree to which snail populations are genetically differentiated from one another.

**Results:**

High levels of snail genetic diversity were found coupled with evidence of geographically-determined population structure but low levels of local inbreeding. The data are consistent with an effect of schistosome infection on population structure of intermediate host snails, but other factors, such as habitat and historical demographic changes, could also be important determinants of the degree of population genetic structure in *Biomphalaria choanomphala*.

**Conclusions:**

The low stratification of populations and high genetic diversity indicates potentially less local compatibility with intermediate snail populations than previously theorized, and highlights the importance of coordinated parasite control strategies across the region.

**Electronic supplementary material:**

The online version of this article (doi:10.1186/s13071-014-0524-4) contains supplementary material, which is available to authorized users.

## Background

Historically, the nature and dynamics of host-parasite relationships has attracted much scientific attention. Parasites of human populations are an area of particular research interest, given the medical interest in controlling their transmission. As such, the genetic diversity and population structure of hermaphroditic *Biomphalaria* freshwater snails of the family Planorbidae*,* which are the obligatory intermediate hosts of the globally important parasitic trematode of humans, *Schistosoma mansoni,* have been the subject of considerable study.

It is known that increased genetic diversity of the host *Biomphalaria* population reduces the overall parasite transmission rate whereas reduced host genetic diversity appears to benefit *S. mansoni* through an overall increase in transmission rate [1-5]. It has also been shown that schistosome infection in *Biomphalaria* reduces snail fitness, but resistance to infection is itself also associated with reduced offspring production, as often seen in other planorbids [6]. As such, it has been predicted that the natural *Biomphalaria* population structure would result in a tightly coupled system whereby parasites and snails co-adapt, resulting in localized compatibility and comparable population structures, but also driving local high diversity [7]. Of course, parasites other than *Schistosoma* could equally be implicated in driving diversity in such a system, as *Biomphalaria* are known to serve as intermediate hosts for a number of nematode and trematode worms [8,9].

Parasites such as *S. mansoni* are, however, not the only external factor known to influence their host. Selection driven by environmental factors, such as seasonality or habitat type, has also been shown to affect freshwater snail population differentiation [10-12]. There may also be anthropogenic influences, for example, removal of snails as part of disease control programs [13].

*B. glabrata* and *B. pfeifferi* are considered two of the most important intermediate host snail species of *S. mansoni* in the New and Old World, respectively, and most previous work on snail-schistosome population structure has focused on these species [14]. There are, however, other regions of the world where intestinal schistosomiasis is highly endemic, yet transmitted by other *Biomphalaria* species [15], and fewer studies have focused on these intermediate hosts. In this study we investigate the genetic diversity and population structure of *Biomphalaria choanomphala* from Lake Victoria, an important lacustrine environment in East Africa, and a well-known regional hot spot for transmission of *S. mansoni* [16-19]. The aim of the study is to determine the population structure of *Biomphalaria choanomphala* in this hyper-endemic region for schistosomiasis, at a large scale, to better understand factors that may contribute to parasite transmission.

## Methods

The degree of population differentiation of *Biomphalaria choanomphala* in Lake Victoria was inferred from genetic variation in two mitochondrial genes (cytochrome oxidase sub-unit one (COI) and 16S ribosomal RNA (16S)), and four bi-parentally inherited nuclear microsatellite loci.

### Sampling methodology and population selection

**Figure 1:**
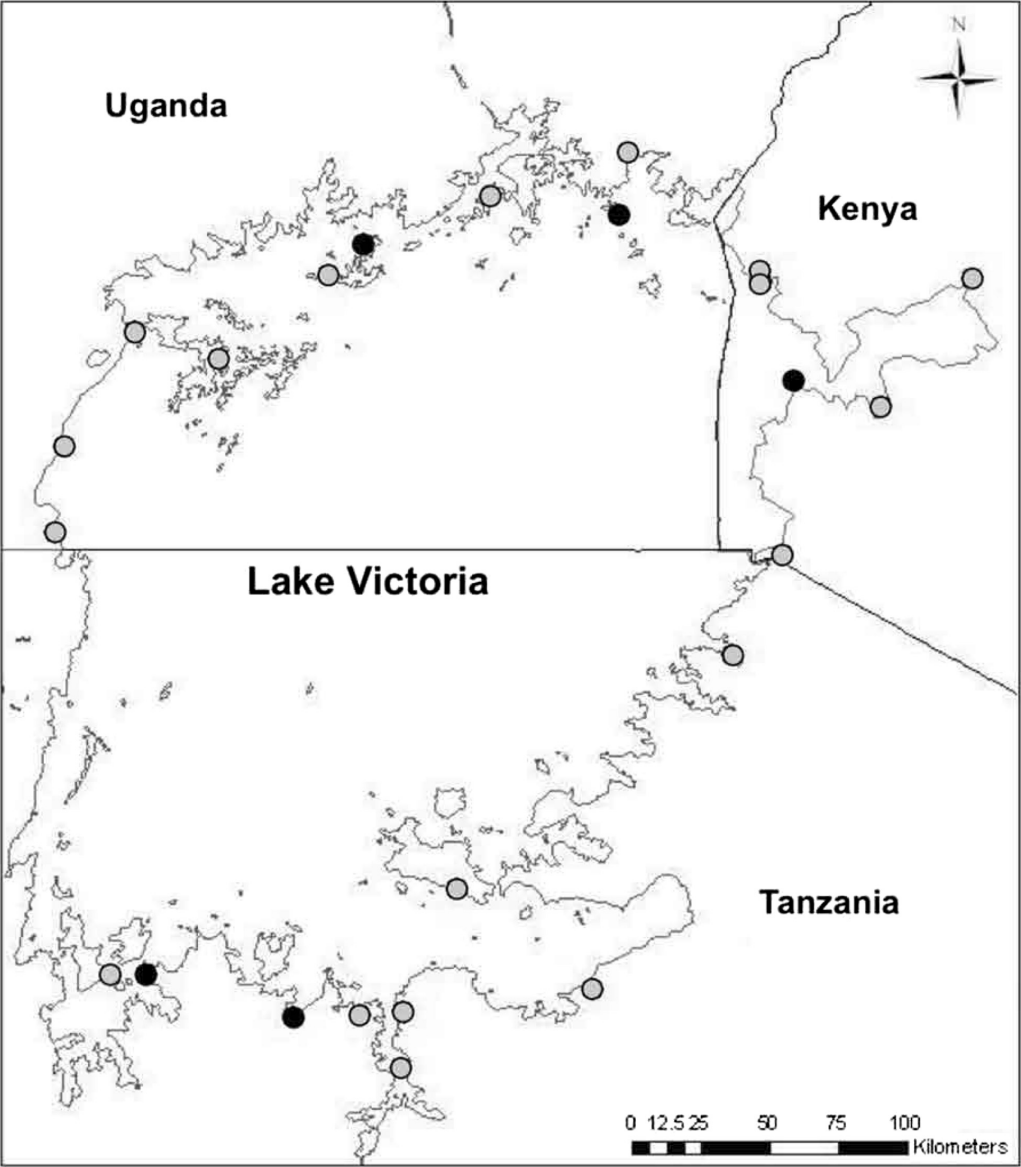
**Map of the 29 study sites around Lake Victoria.** “Single habitat” sites (marked by grey circle) denote an area with a single habitat (lake or marsh); “Joint habitat” sites (marked by black circle) indicate localities where a marsh and lake habitat were found together, and snails collected separately from each.

Snails were collected from 29 sites around the perimeter of Lake Victoria, using hand held scoops (Figure 1). Sampling was semi-quantified, with two collectors each surveying a 50 m length of shoreline for approximately 20 minutes and collecting all *Biomphalaria* found. Sites were selected to ensure an even geographical spread along the lakeshore, in all three countries and in both marsh and lake habitats; five localities were specifically included because they incorporated both habitat types in close proximity. All sites showed signs of human activity near the shoreline. In order to compare sites of similar population density, all sites selected had a high abundance of *Biomphalaria*, defined as 30 or more individuals collected, which accounted for the paucity of sites along the eastern shoreline of the lake in Tanzania, where *Biomphalaria* densities were generally low.

Snails were putatively identified as *B. choanomphala* or *B. sudanica*, based on shell morphology, as these are the species thought primarily to inhabit Lake Victoria [15]. However, recent taxonomic work has suggested that all the *Biomphalaria* in Lake Victoria can be considered a single species of which ecophenotypy of shell form gives rise to the previously considered taxonomic distinctions [20], and thus for the purposes of this study, individuals were simply classed as being one of the two morphotypes of *B. choanomphala*.

All collected snails (in total >30 per site, given the site selection criteria) were exposed, as a group, to sunlight for 2–4 hours to check for infection with *Schistosoma mansoni* or other parasite cercariae [21]. A random sample of snails was examined in this way on subsequent days, to ensure maximum reliability of infection testing. After shedding, snails were placed in glass tubes containing 95% ethanol solution.

### DNA extraction, amplification and sequencing

Genomic DNA was extracted from between 10 and 12 individuals from each of the selected 29 populations, using a standard CTAB extraction [22] with a final re-suspension in pure water. The mitochondrial cytochrome oxidase sub-unit one (COI) gene and the mitochondrial 16S sub-unit of the ribosomal RNA gene were amplified using the Folmer ‘universal’ primers [23] and modified 16ar and 16br primers [24], respectively. The four microsatellite loci were Bpf1, Bpf2, Bpf3 and Bpf10 [25] – all nine of the primers described in referenced paper were tested, but the above four were the only ones which amplified successfully and were polymorphic in the Lake Victoria *Biomphalaria choanomphala* under examination here. Mitochondrial (COI and 16S) amplifications were carried out in 25 μl total volume, with 2.5 μl MgCl_2_ (20 mM concentration), 2.5 μl 5 × buffer, 2.5 μl pre-mixed dNTPs (20 mM concentration), 1 μl each of forward and reverse primer (10 pmol concentration) and one unit of TAQ per reaction. Microsatellite reactions were amplified in 12.5 μl total volume, with half of the volumes stated above, and with forward primer concentration of 50 pmol and reverse (fluorescently labeled) primer concentration of 20 pmol. Cycling conditions in all cases followed published methods [23-25].

Positively amplified products were purified using Millipore PCR_96_ Cleanup kits on a vacuum manifold (Millipore, Billerica, USA) as per manufacturer’s instructions, using pure water for washing and re-suspension. Product concentration was quantified on a Nanodrop ND-1000 Spectrophotometer (Nanodrop Technologies Inc., Willington, USA), and sequencing reactions were performed on mitochondrial purified PCR products using an Applied Biosystems Big Dye Kit (version 1.1) and run on an Applied Biosystems 3730 DNA Analyzer (Applied Biosystems, Carlsbad, USA). Microsatellites were diluted 1:10 in HiDiformamide (Applied Biosystems, as above) and analyzed using an ABI3730 automated sequencer, using a GeneScan 500 LIZ size standard (both Applied Biosystems, as above).

### Population analyses

The overall framework of the population genetics analyses were designed to examine the population structure, diversity, demography, phylogeography and spatial correlations of the *B. choanomphala* populations surveyed from Lake Victoria [2].

COI and 16S mitochondrial sequences were aligned using MUSCLE on the web (http://www.ebi.ac.uk/Tools/msa/muscle/) and edited visually in MacClade v 4 [26]. The aligned sequences were then compared and reduced to unique haplotypes, and the proportion of each haplotype *per* population was recorded. The 16S data contained many gap-rich regions, for which it was impossible to be assured of homology; any region with a gap was therefore removed from the alignment before calculating genetic distances amongst sequences. There was only one indel region in the COI alignment, of a single codon repeat, in three haplotypes, which was therefore easily alignable and retained for haplotype analysis.

Measures of population differentiation were calculated from mitochondrial sequence data using analysis of molecular variance (AMOVA), F-statistics, mismatch analysis, Tajima and Fu’s tests for neutrality and a Mantel z-test for correlation by distance. Each AMOVA was conducted using “country” as the primary grouping, to investigate the level of variation partitioned between each country, versus between populations within each country and within each population. “Country” was deemed to be a potentially important geographical factor given that each country in the region undertakes different levels of schistosomiasis control initiatives, thus potentially influencing the level of parasitism experienced by local snails. All tests were executed in Arlequin v 3.2 [27].

A distance-based, neighbor-joining tree was created in Paup* 4.0 [28] for the unique haplotypes that remained when indels were removed from the alignment from each gene, using the GTR + gamma model of sequence evolution, determined to be the most appropriate model through likelihood testing, also in Paup* 4.0. Median-joining networks were constructed using Network 4.6 [29] based on the gap-free unique haplotypes.

The number and proportion of private microsatellite alleles per population was calculated using the web-based version of Genepop [30]. Calculations of F_ST_ and tests for Hardy-Weinberg equilibrium (through estimation of F_IS_ values per population and per locus) and linkage disequilibrium amongst loci were also done using Genepop. The loci were also tested for evidence of previous bottlenecking events by comparing the allelic diversity against the observed and expected heterozygosity, using the program Bottleneck [31].

## Results

In total, over 300 *Biomphalaria choanomphala* were analyzed from the selected 29 populations. Both the mitochondrial markers and the microsatellites revealed high levels of genetic variation overall, but also significant population structuring throughout the lake as described below.

### Mitochondrial data

The COI and 16S sequences were very genetically variable with 127/308 (CO1) and 181/300(16S) unique haplotypes/total number of sequences respectively. Haplotypes were not highly divergent (0.002-0.058 pairwise distance for COI haplotypes and 0.000-0.047 for 16S). GenBank accession numbers for these datasets are HM769132-HM769258 for COI and HM768950-HM768980 and HM768982-HM769131 for 16S. When unalignable regions with gaps were removed from the analysis of the 16S sequences, the number of unique haplotypes was reduced from 181 to 64. Many common haplotypes were spread throughout the lake, but others were locally restricted and there was high variation in the haplotype richness of the sites: overall, intra-site haplotype diversity estimates varied from 0.200-0.982 for COI and 0.378-1.000 for 16S, where zero indicates complete homogeneity and one indicates maximum diversity.

**Figure 2:**
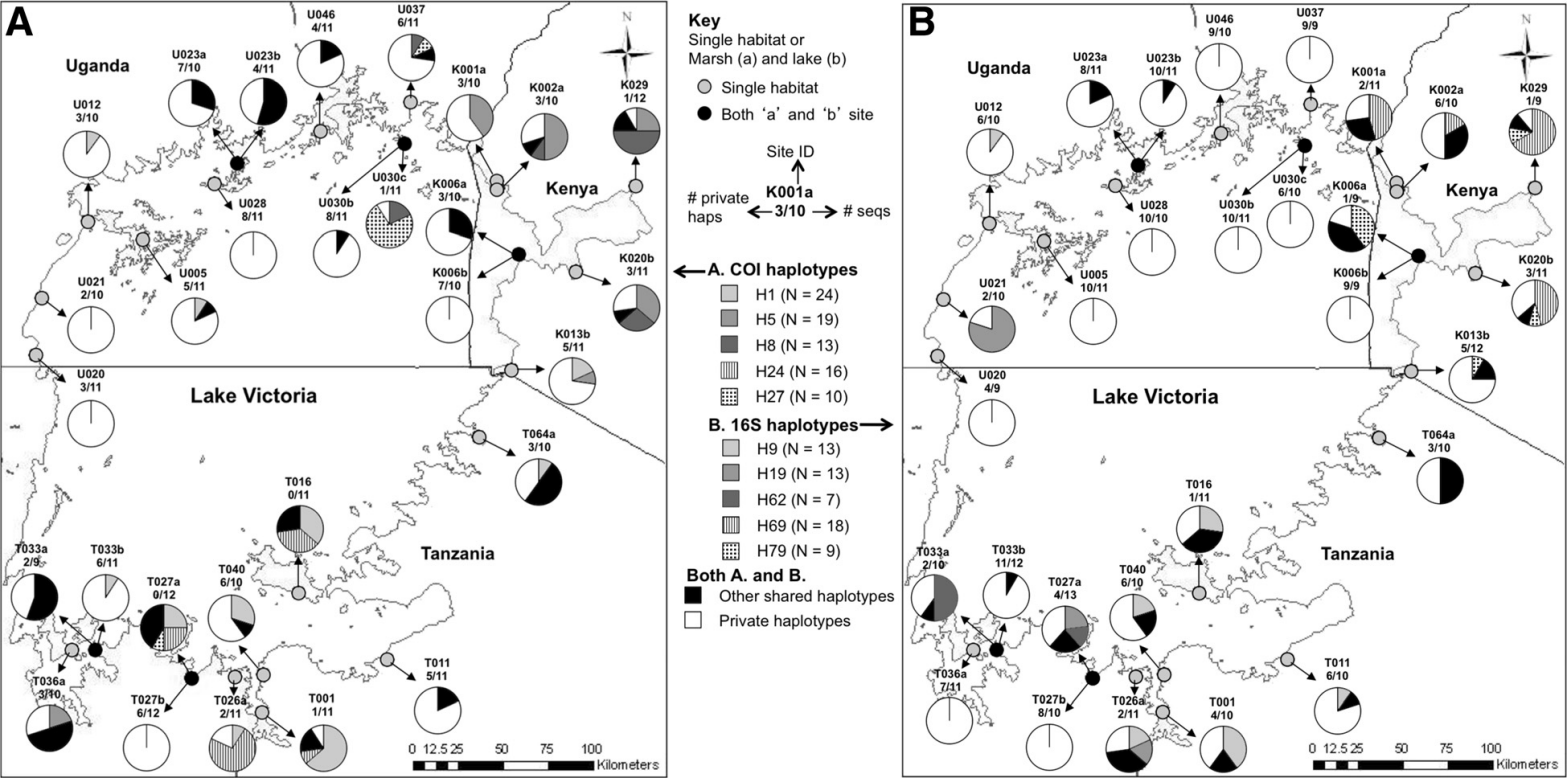
**Distribution of abundant, shared and private haplotypes around Lake Victoria study sites. “A”** shows data for COI haplotypes; **“B”** shows data for 16S haplotypes (with gap dataset).

Figure 2 shows the geographical distribution of the five most abundant COI and 16S (“A” and “B” maps in Figure 2, respectively) haplotypes (without removal of indels) per population across Lake Victoria, as compared to the frequency of other shared haplotypes and “private” haplotypes (i.e. haplotypes only observed at one site). The map reveals patterns of higher and lower haplotype sharing versus uniqueness within and between localities and countries; for example, Kenyan sites tended to share haplotypes whereas Ugandan sites, especially those in the central and western regions of the lakeshore, had high proportions of private haplotypes. Maps showing the distribution of all the haplotypes for COI and 16S, plus a full list of haplotype frequencies per site, can be found in the supplementary information (See Additional file 1: Figure S1, Additional file 2: Figure S2, Additional file 3: Table S1 and Additional file 4: Table S2 for COI and 16S respectively).

**Figure 3:**
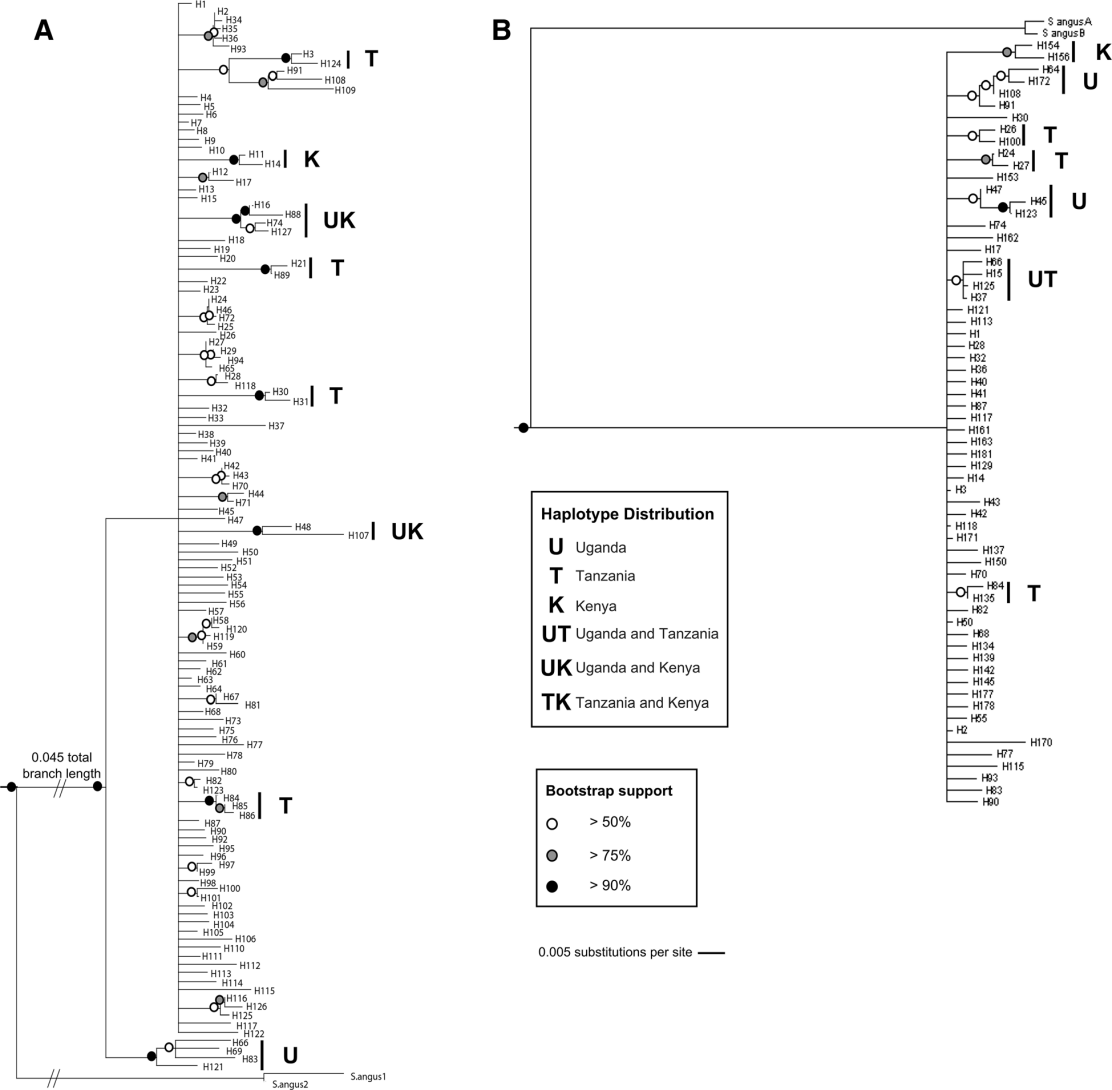
**Neighbor-joining trees of COI (“A”) and 16S (“B”) data.** The level of bootstrap support is indicated by the color of the circle on the node; only support values >50% are shown.

The phylogenetic trees for COI and 16S (Figure 3) were consistent, as would be expected from linked loci, and showed few deep divisions, but several more recent processes of divergence. These supported the hypothesis of geographical structuring, as divergent clades tended to be made up of haplotypes from the same country, or limited to two out of the three countries. The networks for each mitochondrial marker were difficult to analyze visually due to the large number of haplotypes (See Additional file 5: Figure S3 for COI and Additional file 6: Figure S4 for 16S). However there appeared to be evidence of geographical clustering by country; for both markers, the most abundant and geographical widespread haplotype (H1 for COI and H2 for 16S; the network software utilized the gap-free dataset) was a central node within each respective network.

The majority of the variation, for both the COI and the 16S data, was explained at the population level based on the analysis of molecular variance (AMOVA), which sought to determine how diversity was partitioned among and between the different sites. For these data, all three levels of structure (intergroup, intragroup and intrapopulation) were statistically significantly differentiated (see Table 1).

**Table 1:** **Analysis of molecular variance (AMOVA) results for COI and 16S data**

	**Source of variation**	**Degrees of freedom**	**Sum of squares**	**Variance components**	**Percentage of variation**	**p-value**
**COI sequences**	**Among countries**	2	173.35	0.6646	12.75	< 0.0001
	**Among populations, within countries**	26	512.68	1.5774	30.26	< 0.0001
	**Within populations**	279	829.10	2.9717	57.00	< 0.0001
	**Total**	307	1515.12	5.2137	100.00	
**16S sequences**	**Among countries**	2	155.88	0.5893	10.00	< 0.001
	**Among populations, within countries**	26	524.77	1.5935	27.04	< 0.0001
	**Within populations**	271	1005.55	3.7105	62.96	< 0.0001
	**Total**	299	1686.20	5.8933	100.00	

When pair-wise fixation index (F_ST_) values were calculated between each of the populations in turn, the majority demonstrated significant levels of differentiation, for both types of molecular marker. The range in values was 0.000-0.921 for COI and 0.000-0.845 for 16S. For the 16S data, all but one of the non-significant F_ST_ values were between sites in the same country, once again demonstrating geographically defined structuring of the populations for this gene. All but one paired site showed significant differentiation between the populations from lake versus marsh habitat, indicating even small-scale geographical segregation. Full tables of the pairwise F_ST_ values and their significance can be found in the supplementary information (See Additional file 7: Tables S3 and Additional file 8: Tables S4 respectively for COI and 16S).

All of the populations conformed to the assumptions of the neutral hypothesis, as tested for by Tajima’s D statistic for the 16S data; one population, T001, deviated significantly (p <0.001) from assumptions of normality when the COI data was analyzed. However, this population also had a significant result for the sum of squares mismatch analysis (p <0.001), suggesting that demographic change, such as population expansion, may have biased the neutrality test. No other populations, for either marker, came out as significant in the mismatch analysis.

The relationship with geography was elucidated through the significance of the Mantel z-test for both the COI and 16S data (p =0.001, for both), meaning that the genetic and geographical distances between the populations was positively correlated; in other words, sites closer together were more likely to be genetically similar, even if the composition of distinct haplotypes differed per site (as seen in Figure 3 and Additional file 1: Figure S1). This demonstrated an overall relationship between genetic differentiation and geographic distance, on top of the localized differentiation already described, such as between marsh and lake habitats (Additional file 5: Figure S3 and Additional file 6: Figure S4).

### Microsatellite data

The microsatellite data were tested for departures from Hardy-Weinberg equilibrium (HWE), linkage disequilibrium and for an excess of heterozygosity relative to allelic diversity, which would be consistent with populations having recently passed through a bottleneck.

As with the mitochondrial data, based on an AMOVA, the majority of the variation in microsatellite genotype was explained at the population level; values of differentiation at the intragroup and intrapopulation were both highly significant (explaining 10.55% and 89.13% of the total variation observed, respectively: Table 2). Much less variation was explained between country-level groups for the microsatellite data (0.31%) than for either of the mitochondrial genes, but instead there was a larger proportion of diversity within populations (>89%). Pairwise F_ST_ values ranged from 0.000-0.298, but fewer values were significant between populations, compared to the mitochondrial data. The full matrix of pairwise F_ST_ values and their significance can be found in the supplementary information (Additional file 9: Table S5). Supporting the F_ST_ values in suggesting low gene flow between populations, 17 out of the 28 populations with microsatellite data had private alleles at one or more loci, and the proportions of private alleles across all populations and alleles was highly significant (Fisher’s method, p = <0.001). The microsatellite data also supported the mitochondrial evidence for genetic spatial autocorrelation, as the Mantel z-test was significant (p = 0.017).

**Table 2:** **Analysis of molecular variance (AMOVA) results for microsatellite data**

	**Source of variation**	**Degrees of freedom**	**Sum of squares**	**Variance components**	**Percentage of variation**	**p -value**
**Microsatellite data**	**Among countries**	2	9.51	0.0057	0.31	0.237
	**Among populations, within countries**	23	88.36	0.1900	10.55	< 0.0001
	**Within populations**	286	459.05	1.6051	89.13	< 0.0001
	**Total**	311	556.93	1.8008	99.99	

Intrapopulation tests however showed low levels of inbreeding within populations, based on the calculations of F_IS_ values per locus as well as across all loci. F_IS_ values, across all loci, per population, ranged from −0.186 to 0.168 (Table 3), with a mean value across all populations of 0.019, indicative of random mating. More generally, all populations conformed to the assumptions of HWE in terms of observed and expected heterozygosity, supporting the hypothesis that there are low levels of selfing.

**Table 3:** **Diversity estimates per site for COI, 16S and microsatellite data**

**Site**	**General information**		**COI sequences**		**16S sequences**		**Microsatellite data**		
	**Shedding**	**Habitat**	**Gene diversity**	**Nucleotide diversity**	**Gene diversity**	**Nucleotide diversity**	**Average gene div.**	**Private alleles**	**F**_**IS**_
**K001a**	N	Marsh	0.836	0.005	0.746	0.005	0.885	0.000	−0.010
**K002a**	N	Marsh	0.712	0.001	0.978	0.011	0.878	0.029	−0.001
**K006a**	N	Marsh	0.855	0.014	0.500	0.005	0.790	0.079	−0.077
**K006b**	N	Lake	0.889	0.026	1.000	0.043	0.843	0.000	0.119
**K013b**	N	Lake	0.909	0.014	0.879	0.012	0.710	0.139	0.025
**K020b**	N	Lake	0.933	0.004	0.800	0.006	0.825	0.000	0.083
**K029**	N	Lake	0.818	0.015	0.583	0.004	0.828	0.000	0.034
**T001**	Y	Marsh	0.978	0.017	0.867	0.017	0.754	0.016	−0.186
**T011**	N	Lake	0.927	0.009	0.933	0.016	0.738	0.015	0.073
**T016**	N	Marsh	0.964	0.016	0.727	0.008	0.743	0.000	−0.077
**T026a**	N	Marsh	0.964	0.009	0.891	0.005	0.636	0.019	−0.112
**T027a**	N	Marsh	0.782	0.010	0.923	0.018	0.848	0.014	−0.180
**T027b**	N	Lake	0.733	0.003	0.956	0.033	0.735	0.050	0.038
**T033a**	N	Marsh	0.778	0.002	0.711	0.014	0.738	0.000	0.026
**T033b**	Y	Lake	0.778	0.004	1.000	0.021	0.758	0.014	0.134
**T036a**	N	Marsh	0.618	0.008	0.818	0.016	0.744	0.013	0.092
**T040**	N	Lake	0.727	0.003	0.978	0.011	0.803	0.033	0.168
**T064a**	N	Marsh	0.491	0.002	0.889	0.022	0.783	0.000	0.112
**U005**	N	Lake	0.864	0.011	0.982	0.027	0.819	0.013	0.101
**U012**	N	Marsh	0.639	0.010	0.911	0.020	0.845	0.000	0.058
**U020**	N	Marsh	0.756	0.006	0.583	0.007	0.806	0.028	0.116
**U021**	N	Marsh	0.844	0.015	0.378	0.002	0.754	0.000	−0.119
**U023a**	N	Lake	0.644	0.004	0.978	0.033	0.818	0.000	−0.039
**U023b**	Y	Marsh	0.346	0.004	1.000	0.028	0.878	0.016	0.109
**U028**	Y	Lake	0.200	0.000	1.000	0.025	0.857	0.015	0.014
**U030b**	N	Marsh	0.982	0.016	0.982	0.028	0.877	0.000	−0.104
**U030c**	N	Lake	0.473	0.002	0.844	0.014	NA	NA	NA
**U037**	Y	Lake	0.836	0.005	0.978	0.019	0.833	0.012	0.091
**U046**	N	Lake	0.712	0.001	1.000	0.030	0.868	0.025	0.050

Information on infection status with schistosomes and the habitat type are also given. “N” stands for “No” and “Y” represents “Yes”. “div.”stands for diversity and “FIS” indicates the inbreeding co-efficient.

There was also no evidence for linkage disequilibrium between the loci, suggesting that the alleles considered are not closely associated in the genome and are mixing randomly during gamete production. Four populations (K013b, T001, T027b and U028), when tested for evidence of a past bottleneck, showed heterozygote excess (p = 0.031 for all four), but this value was not significant once the Bonferroni correction for multiple tests was applied.

## Discussion

### *Population structure of* Biomphalaria choanomphala *in Lake Victoria*

The populations studied here were characterized by high intrapopulation diversity as well as high levels of population structure, with low levels of gene flow as inferred from molecular data and negligible inbreeding. High diversity is consistent with what would be expected if parasitism were strongly influencing population structure [3,7], and the low F_IS_ values and apparent random mating observed here further supports this hypothesis. The snails collected for this research were examined for infection with schistosomes and other parasites in the field as part of a different study; however, without more accurate and consistent methods such as molecular probes [32] for the detection of infected snails (and indeed, infection with potentially more than one parasite species [33]) and infected snail populations, it is difficult to determine whether parasitism is indeed a key driver of population structure in this system. Similarly, future studies should seek to use genetic markers which are known to correlate to parasite resistance or susceptibility, which were not available when this study was conducted, but have since been identified in several studies [34,35]. Even so, the challenges of modifying markers for use on field populations of less well-studied African *Biomphalaria* remain.

We would also expect parasite populations to adapt to local snails, thus mirroring to some extent their population structure. We used a Mantel test [36] to compare the population structure of *B. choanomphala* from the six sites from which there were published data on schistosome population structure [19], and found no correlation. Notwithstanding the limitation of the small number of sites, this conforms to other studies where parasite populations were much less differentiated than those of the intermediate host, suggesting local compatibility in this context is being overshadowed by other factors such as high parasite migration [36,37]. Definitive host migration has been put forward as a factor that might homogenize parasite populations across a wide area, and this is compatible with the Lake Victoria context, where human populations are known to move widely throughout the region [18].

### Role of other factors influencing population structure

Given the possibility of parasitism as a driver for population structure in Lake Victoria snail populations, it is important to consider what other factors might be influential in this system, and particularly how these differ from previous studies of *Biomphalaria* in other settings. Such considerations link back to parasitism, and particularly control of schistosomiasis in human populations, in terms of gaining a better understanding of drivers for distribution and abundance of host snails under different conditions. Studies of *B. pfeifferi* in Madagascar, for example, revealed high levels of interpopulation variation but, in contrast to what we observed here, consistently low levels of intrapopulation genetic differentiation [11]. This was explained as being due to the combination of low levels of migration combined with habitat stochasticity; *B. pfeifferi* are also known to be frequent in-breeders, which would assist in maintaining a metapopulation with seasonal local extinction events [38].

In contrast, Lake Victoria possesses very different environmental conditions; as a very large, permanent lake, it rarely experiences the kind of environmental perturbations that characterize temporally transient ponds and streams. Such homogeneity of habitat, with associated infrequent extinctions and opportunity for genetic drift, could account for the high levels of intrapopulation diversity that are maintained in Lake Victorian *B. choanomphala* [2]. This applies mainly to the lake habitats; indeed, this study showed significantly lower levels of gene and nucleotide diversity in the marsh habitats. However, in both lake and marsh populations, the tests for bottleneck events were uniformly negative, suggesting that sudden demographical events are not overwhelmingly contributing to the patterns of diversity of Lake Victoria *Biomphalaria.*

### Effect on transmission of intestinal schistosomiasis

Although the low population structure in *S. mansoni* observed previously in this region counters the hypothesis of highly specialized local parasite compatibility [19] for both host and parasite, certain *B. choanomphala* haplotypes were widespread around the lake. This suggests that, as seen in *B. pfeifferi* from elsewhere in Africa, there may be a risk of transmissive genotypes becoming widely successful, if there is selection for traits independent of parasite susceptibility [39].

Another factor to consider is human-mediated changes to parasite populations: all three countries bordering Lake Victoria have initiated efforts to control schistosomiasis in human populations through mass treatment campaigns, although these initiatives are not synchronized and differ in their degree of coverage. The low levels of population structuring seen in the parasite [19] are likely a function of high terminal host migration and rapid dispersal of the parasite into novel localities [36,40]; if some populations of *S. mansoni* experience more intense selection pressure based on frequent mass drug administration campaigns, and given the generally transmissive snail populations, there is a risk that these highly adapted forms of *S. mansoni* will be able to spread rapidly throughout the region. This emphasizes the need for coordinated control strategies, both at the level of human treatment as well as improved water access and sanitation, throughout lakeshore communities in the region.

## Conclusions

In summary, our analysis observed high genetic diversity of *B choanomphala* snails, yet also high population structuring; the high levels of observed inter- and intrapopulation diversity are consistent with parasitism as an influencing factor, but further investigation is needed, utilizing new tools to detect infection and new markers directly associated with resistance or susceptibility, in order to confirm the role of parasitism in driving high diversity in this system. Moreover, other factors, such as environmental and demographical stability may also contribute to the observed population structure. From a public health perspective, population genetic surveys of intermediate hosts should seek to increase the scale of their focus, as parasite transmission is likely influenced by factors, such as human migration and national treatment campaigns, that act on a national or even regional level. Public health initiatives should take such issues of scale into consideration when designing control strategies.

## Additional files

Additional file 1: Figure S1. Haplotype map of all COI haplotypes observed. Additional file 2: Figure S2. Haplotype map of all 16S haplotypes observed. Additional file 3: Table S1. List of all COI haplotypes and their frequencies, per site. Additional file 4: Table S2. List of all 16S haplotypes and their frequencies, per site. Additional file 5: Figure S3. Median-joining network of COI haplotypes. Additional file 6: Figure S4. Median-joining network of 16S haplotypes (without-gap haplotypes). Additional file 7: Table S3. Full table of pairwise F_ST_ values per site for COI marker. Non-significant pairwise distances are given in italics. Additional file 8: Table S4. Full table of pairwise F_ST_ values per site for 16S marker. Non-significant pairwise distances are given in italics. Additional file 9: Table S5. Full table of pairwise F_ST_ values per site for microsatellite markers. Non-significant pairwise distances are given in italics.
