# Supplementary figures and images for: The population genetic structure of Biomphalaria choanomphala in Lake Victoria, East Africa: implications for schistosomiasis transmission
Source: Parasit Vectors. 2014 Nov 19;7:524. doi: 10.1186/s13071-014-0524-4 (PMC4254209; doi:10.1186/s13071-014-0524-4)

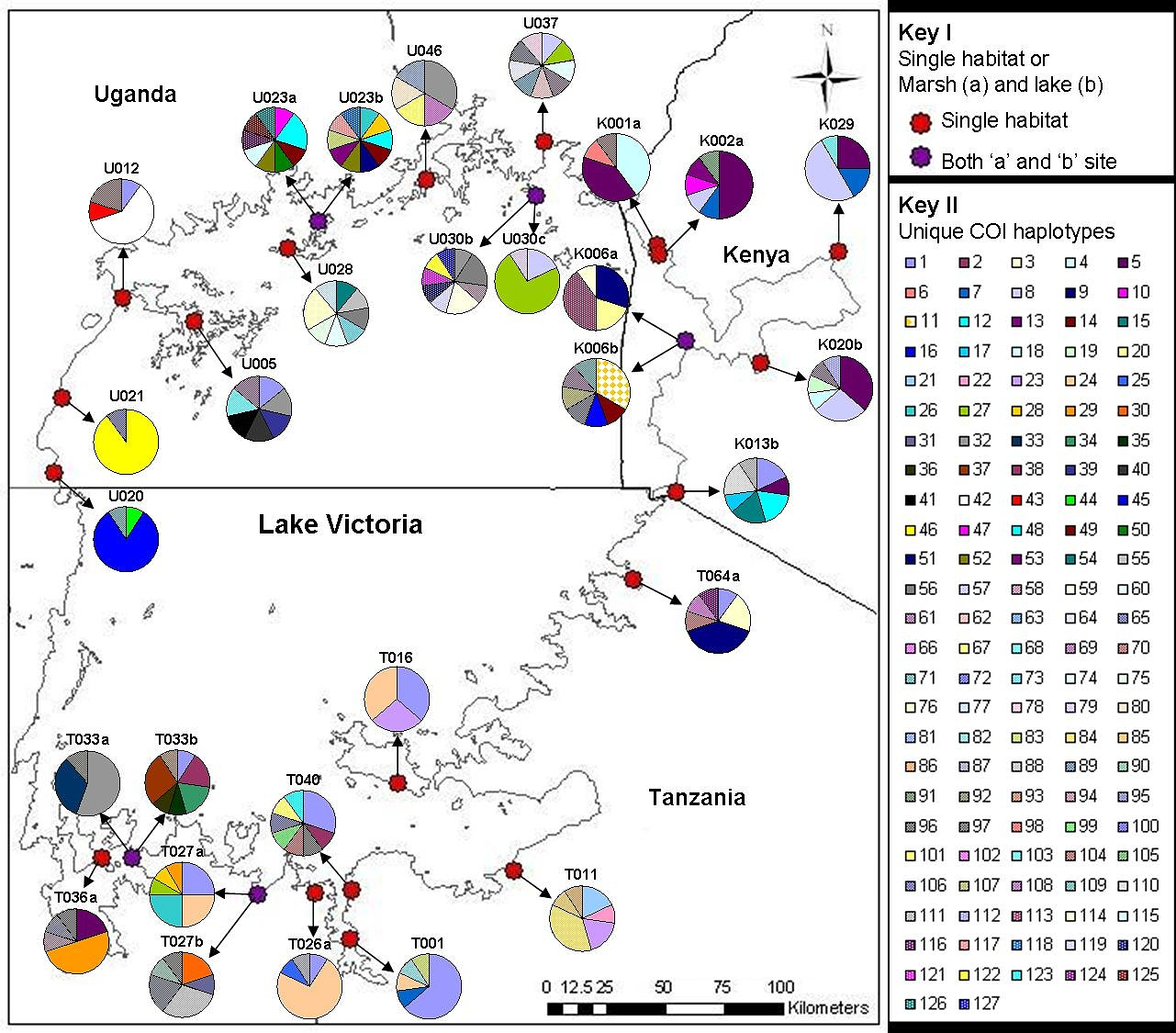

Supplement: Additional file 1: Figure S1. — Haplotype map of all COI haplotypes observed. [file 13071_2014_524_MOESM1_ESM.jpeg]

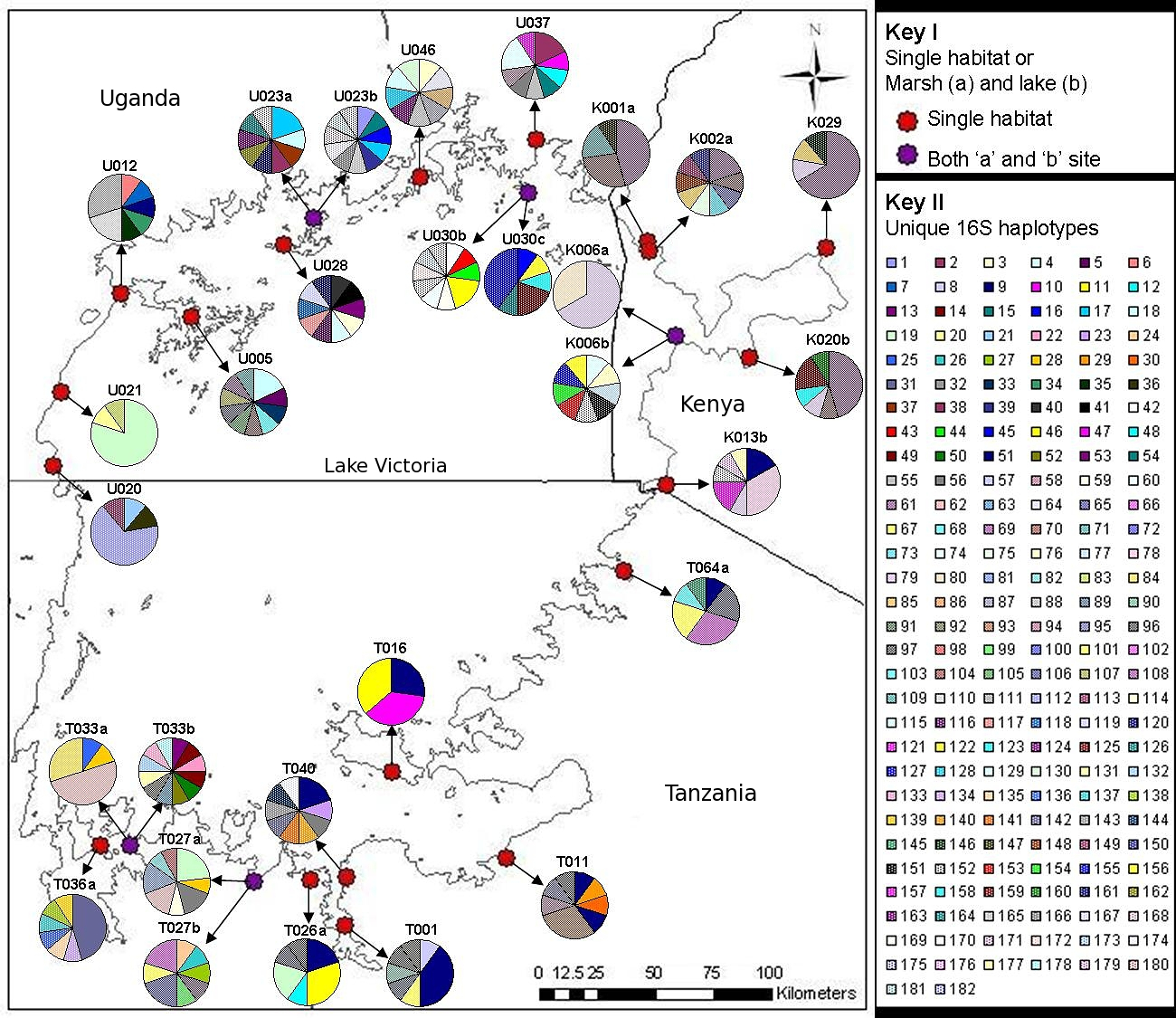

Supplement: Additional file 2: Figure S2. — Haplotype map of all 16S haplotypes observed. [file 13071_2014_524_MOESM2_ESM.jpeg]

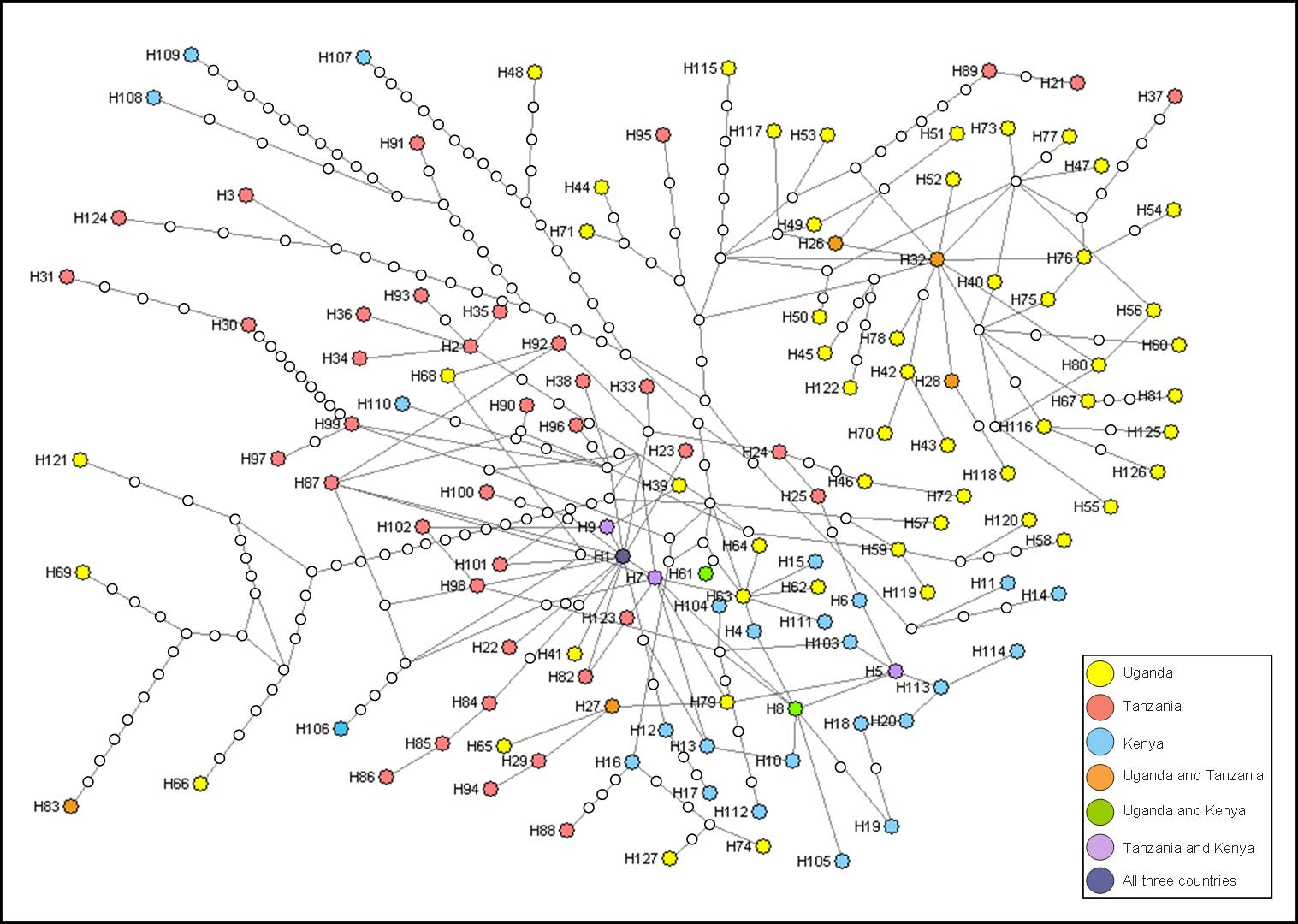

Supplement: Additional file 5: Figure S3. — Median-joining network of COI haplotypes. [file 13071_2014_524_MOESM5_ESM.jpeg]

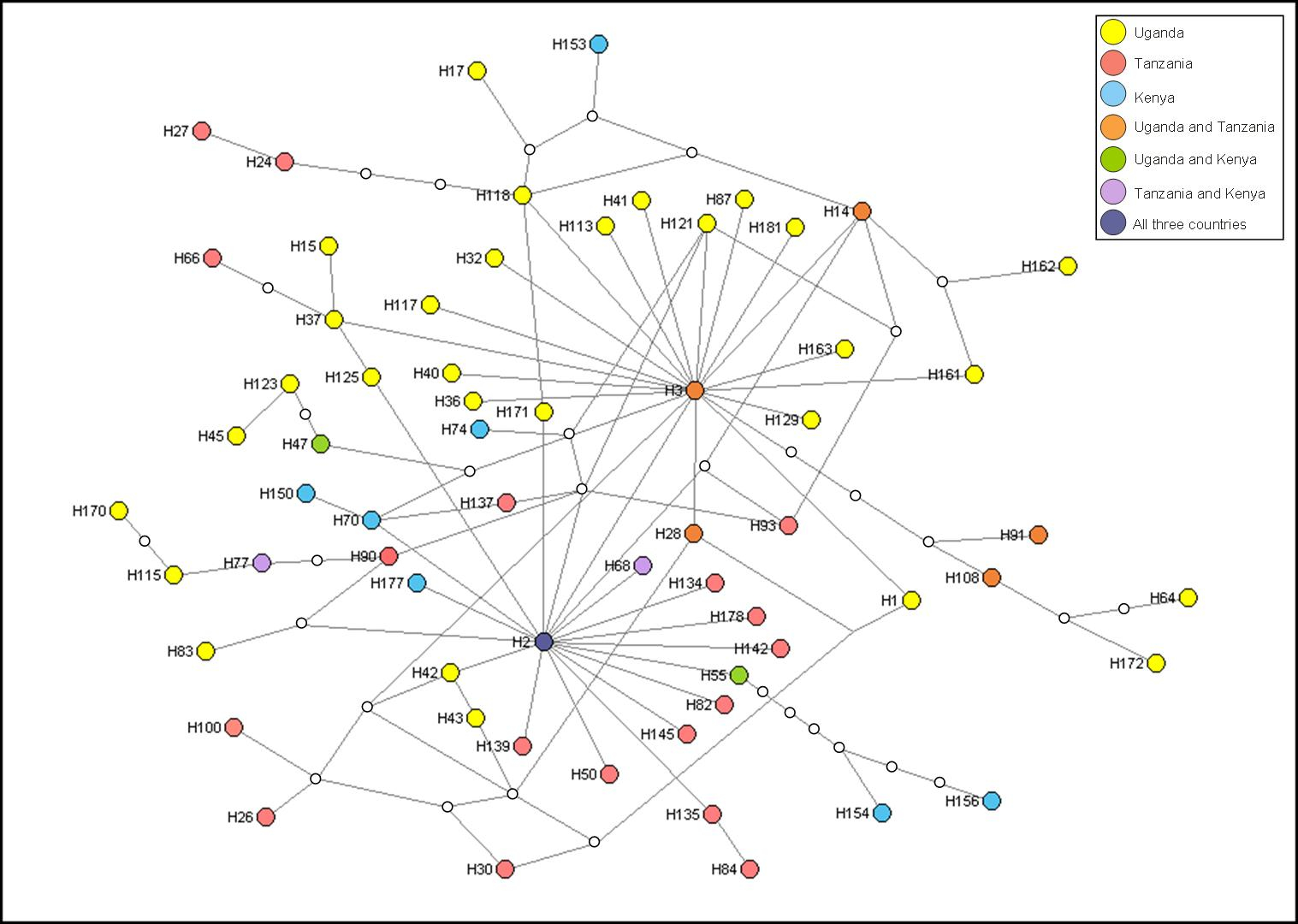

Supplement: Additional file 6: Figure S4. — Median-joining network of 16S haplotypes (without-gap haplotypes). [file 13071_2014_524_MOESM6_ESM.jpeg]
