# Supplementary material for: The population genetic structure of Biomphalaria choanomphala in Lake Victoria, East Africa: implications for schistosomiasis transmission
Source: Parasit Vectors. 2014 Nov 19;7:524. doi: 10.1186/s13071-014-0524-4 (PMC4254209; doi:10.1186/s13071-014-0524-4)
Supplement: Additional file 3: Table S1. — List of all COI haplotypes and their frequencies, per site. [file 13071_2014_524_MOESM3_ESM.docx]

| **Site** | **Haplotypes** | **Frequency** | **Site** | **Haplotypes** | **Frequency** |
| --- | --- | --- | --- | --- | --- |
| K001a | 4 | 4 | T001 | 1 | 7 |
|  | 5 | 4 |  | 7 | 1 |
|  | 6 | 1 |  | 24 | 1 |
|  | 104 | 1 |  | 82 | 1 |
| K002a | 5 | 5 |  | 83 | 1 |
|  | 7 | 1 | T011 | 21 | 2 |
|  | 8 | 1 |  | 22 | 1 |
|  | 10 | 1 |  | 23 | 2 |
|  | 13 | 1 |  | 84 | 4 |
|  | 105 | 1 |  | 85 | 1 |
| K006a | 9 | 3 |  | 86 | 1 |
|  | 20 | 2 | T016 | 1 | 4 |
|  | 113 | 4 |  | 23 | 3 |
|  | 114 | 1 |  | 24 | 4 |
| K006b | 11 | 3 | T026a | 1 | 1 |
|  | 14 | 1 |  | 24 | 8 |
|  | 16 | 2 |  | 25 | 1 |
|  | 106 | 1 |  | 87 | 1 |
|  | 107 | 1 | T027a | 1 | 3 |
|  | 108 | 1 |  | 24 | 3 |
|  | 109 | 1 |  | 26 | 3 |
| K013b | 1 | 2 |  | 27 | 1 |
|  | 5 | 1 |  | 28 | 1 |
|  | 12 | 2 |  | 29 | 1 |
|  | 15 | 2 | T027b | 30 | 2 |
|  | 17 | 1 |  | 31 | 1 |
|  | 110 | 2 |  | 88 | 3 |
|  | 111 | 1 |  | 89 | 2 |
| K020b | 5 | 4 |  | 90 | 1 |
|  | 8 | 3 |  | 91 | 1 |
|  | 18 | 1 | T033a | 32 | 5 |
|  | 19 | 1 |  | 33 | 3 |
|  | 61 | 1 |  | 92 | 1 |
|  | 112 | 1 | T033b | 1 | 1 |
| K029 | 5 | 3 |  | 2 | 2 |
|  | 7 | 2 |  | 34 | 2 |
|  | 8 | 6 |  | 35 | 1 |
|  | 103 | 1 |  | 36 | 1 |
|  |  |  |  | 37 | 3 |
|  |  |  |  | 93 | 1 |

| **Site** | **Haplotypes** | **Frequency** | **Site** | **Haplotypes** | **Frequency** |
| --- | --- | --- | --- | --- | --- |
| T036a | 5 | 2 | U023b | 26 | 1 |
|  | 29 | 5 |  | 28 | 1 |
|  | 94 | 1 |  | 48 | 1 |
|  | 95 | 1 |  | 49 | 1 |
|  | 96 | 1 |  | 51 | 2 |
| T040 | 1 | 3 |  | 52 | 1 |
|  | 38 | 1 |  | 53 | 1 |
|  | 97 | 1 |  | 83 | 1 |
|  | 98 | 1 |  | 117 | 1 |
|  | 99 | 1 |  | 118 | 1 |
|  | 100 | 1 | U028 | 54 | 1 |
|  | 101 | 1 |  | 55 | 1 |
|  | 123 | 1 |  | 56 | 3 |
| T064a | 1 | 1 |  | 73 | 1 |
|  | 3 | 2 |  | 74 | 1 |
|  | 9 | 4 |  | 75 | 1 |
|  | 98 | 1 |  | 76 | 2 |
|  | 102 | 1 |  | 77 | 1 |
|  | 124 | 1 | U030b | 32 | 1 |
| U005 | 1 | 1 |  | 57 | 2 |
|  | 32 | 1 |  | 58 | 1 |
|  | 39 | 1 |  | 59 | 2 |
|  | 40 | 5 |  | 119 | 1 |
|  | 41 | 1 |  | 120 | 1 |
|  | 68 | 1 |  | 121 | 1 |
|  | 69 | 1 |  | 122 | 1 |
| U012 | 1 | 1 |  | 127 | 1 |
|  | 42 | 6 | U030c | 8 | 2 |
|  | 43 | 1 |  | 27 | 8 |
|  | 70 | 2 |  | 79 | 1 |
| U020 | 44 | 1 | U037 | 8 | 1 |
|  | 45 | 9 |  | 27 | 1 |
|  | 71 | 1 |  | 60 | 1 |
| U021 | 46 | 9 |  | 61 | 1 |
|  | 72 | 1 |  | 62 | 2 |
| U023a | 47 | 1 |  | 63 | 1 |
|  | 48 | 2 |  | 64 | 2 |
|  | 49 | 1 |  | 65 | 1 |
|  | 50 | 1 |  | 78 | 1 |
|  | 52 | 1 | U046 | 32 | 2 |
|  | 115 | 1 |  | 66 | 1 |
|  | 116 | 1 |  | 67 | 5 |
|  | 125 | 1 |  | 80 | 2 |
|  | 126 | 1 |  | 81 | 1 |
|  |  |  |  |  |  |
