# Supplementary material for: The population genetic structure of Biomphalaria choanomphala in Lake Victoria, East Africa: implications for schistosomiasis transmission
Source: Parasit Vectors. 2014 Nov 19;7:524. doi: 10.1186/s13071-014-0524-4 (PMC4254209; doi:10.1186/s13071-014-0524-4)
Supplement: Additional file 4: Table S2. — List of all 16S haplotypes and their frequencies, per site. [file 13071_2014_524_MOESM4_ESM.docx]

| **Site** | **Haplotype** | **Frequency** | **Site** | **Haplotype** | **Frequency** | **Site** | **Haplotype** | **Frequency** |
| --- | --- | --- | --- | --- | --- | --- | --- | --- |
| K001a | 69 | 5 | T001 | 8 | 1 | T033a | 25 | 1 |
|  | 70 | 3 |  | 9 | 4 |  | 28 | 1 |
|  | 71 | 2 |  | 67 | 1 |  | 62 | 5 |
|  | 147 | 1 |  | 89 | 1 |  | 84 | 3 |
| K002a | 68 | 2 |  | 90 | 1 | T033b | 13 | 1 |
|  | 70 | 1 |  | 91 | 1 |  | 14 | 1 |
|  | 72 | 1 |  | 92 | 1 |  | 22 | 1 |
|  | 73 | 1 | T011 | 9 | 1 |  | 49 | 1 |
|  | 75 | 1 |  | 29 | 1 |  | 50 | 1 |
|  | 85 | 1 |  | 30 | 1 |  | 52 | 1 |
|  | 148 | 1 |  | 51 | 1 |  | 63 | 1 |
|  | 149 | 1 |  | 93 | 3 |  | 92 | 1 |
|  | 150 | 1 |  | 94 | 1 |  | 131 | 1 |
| K006a | 79 | 6 |  | 95 | 1 |  | 132 | 1 |
|  | 80 | 3 |  | 96 | 1 |  | 133 | 1 |
| K006b | 74 | 1 | T016 | 9 | 3 |  | 178 | 1 |
|  | 76 | 1 |  | 10 | 4 | T036a | 31 | 5 |
|  | 77 | 1 |  | 11 | 4 |  | 134 | 1 |
|  | 151 | 1 | T026a | 9 | 2 |  | 135 | 1 |
|  | 152 | 1 |  | 11 | 3 |  | 136 | 1 |
|  | 153 | 1 |  | 12 | 1 |  | 137 | 1 |
|  | 154 | 1 |  | 19 | 2 |  | 138 | 1 |
|  | 155 | 1 |  | 65 | 1 |  | 139 | 1 |
|  | 156 | 1 |  | 97 | 2 | T040 | 9 | 2 |
| K013b | 51 | 2 | T027a | 19 | 3 |  | 23 | 1 |
|  | 78 | 4 |  | 28 | 1 |  | 57 | 1 |
|  | 79 | 1 |  | 57 | 2 |  | 140 | 1 |
|  | 157 | 2 |  | 59 | 1 |  | 141 | 1 |
|  | 175 | 1 |  | 62 | 2 |  | 142 | 1 |
|  | 176 | 1 |  | 81 | 2 |  | 143 | 1 |
|  | 177 | 1 |  | 82 | 1 |  | 144 | 1 |
| K020b | 69 | 5 |  | 98 | 1 |  | 174 | 1 |
|  | 70 | 1 | T027b | 24 | 1 | T064a | 51 | 1 |
|  | 79 | 1 |  | 26 | 1 |  | 65 | 2 |
|  | 158 | 1 |  | 27 | 1 |  | 66 | 3 |
|  | 159 | 2 |  | 61 | 1 |  | 67 | 2 |
|  | 160 | 1 |  | 99 | 1 |  | 68 | 1 |
| K029 | 69 | 6 |  | 100 | 2 |  | 145 | 1 |
|  | 79 | 1 |  | 101 | 1 |  |  |  |
|  | 85 | 1 |  | 102 | 2 |  |  |  |
|  | 146 | 1 |  |  |  |  |  |  |

| **Site** | **Haplotype** | **Frequency** | **Site** | **Haplotype** | **Frequency** |
| --- | --- | --- | --- | --- | --- |
| U005 | 4 | 2 | U028 | 40 | 1 |
|  | 5 | 1 |  | 41 | 1 |
|  | 33 | 1 |  | 53 | 1 |
|  | 103 | 1 |  | 114 | 1 |
|  | 104 | 1 |  | 115 | 1 |
|  | 105 | 1 |  | 116 | 1 |
|  | 106 | 1 |  | 117 | 1 |
|  | 107 | 1 |  | 118 | 1 |
|  | 108 | 1 |  | 119 | 1 |
|  | 109 | 1 |  | 120 | 1 |
| U012 | 6 | 1 | U030b | 42 | 1 |
|  | 7 | 1 |  | 43 | 1 |
|  | 9 | 1 |  | 44 | 1 |
|  | 34 | 1 |  | 46 | 2 |
|  | 35 | 1 |  | 169 | 1 |
|  | 110 | 2 |  | 170 | 1 |
|  | 111 | 3 |  | 171 | 1 |
| U020 | 21 | 1 |  | 172 | 1 |
|  | 36 | 1 |  | 173 | 1 |
|  | 112 | 6 |  | 180 | 1 |
|  | 113 | 1 | U037 | 2 | 1 |
| U021 | 19 | 8 |  | 47 | 1 |
|  | 20 | 1 |  | 48 | 1 |
|  | 83 | 1 |  | 54 | 1 |
| U023a | 17 | 2 |  | 55 | 1 |
|  | 18 | 2 |  | 56 | 1 |
|  | 37 | 1 |  | 58 | 1 |
|  | 38 | 1 |  | 60 | 2 |
|  | 161 | 1 |  | 121 | 1 |
|  | 162 | 1 | U046 | 3 | 1 |
|  | 163 | 1 |  | 64 | 1 |
|  | 164 | 1 |  | 86 | 1 |
|  | 179 | 1 |  | 87 | 1 |
| U023b | 1 | 1 |  | 88 | 1 |
|  | 15 | 1 |  | 124 | 1 |
|  | 16 | 1 |  | 128 | 1 |
|  | 17 | 1 |  | 129 | 1 |
|  | 39 | 1 |  | 130 | 1 |
|  | 165 | 1 |  |  |  |
|  | 166 | 1 |  |  |  |
|  | 167 | 1 |  |  |  |
|  | 168 | 1 |  |  |  |
|  | 181 | 1 |  |  |  |
|  | 182 | 1 |  |  |  |
