# Supplementary material for: The population genetic structure of Biomphalaria choanomphala in Lake Victoria, East Africa: implications for schistosomiasis transmission
Source: Parasit Vectors. 2014 Nov 19;7:524. doi: 10.1186/s13071-014-0524-4 (PMC4254209; doi:10.1186/s13071-014-0524-4)
Supplement: Additional file 7: Table S3. — Full table of pairwise FST values per site for COI marker. Non-significant pairwise distances are given in italics. [file 13071_2014_524_MOESM7_ESM.doc]

| **Site** | **K001a** | **K002a** | **K006a** | **K006b** | **K013b** | **K020b** | **K029** | **T001** | **T011** | **T016** | **T026a** | **T027a** | **T027b** | **T033a** |
| --- | --- | --- | --- | --- | --- | --- | --- | --- | --- | --- | --- | --- | --- | --- |
| **K001a** | * |  |  |  |  |  |  |  |  |  |  |  |  |  |
| **K002a** | *0.0719* | * |  |  |  |  |  |  |  |  |  |  |  |  |
| **K006a** | 0.2471 | 0.2166 | * |  |  |  |  |  |  |  |  |  |  |  |
| **K006b** | 0.3481 | 0.3532 | 0.3665 | * |  |  |  |  |  |  |  |  |  |  |
| **K013b** | 0.2904 | 0.2624 | 0.2846 | 0.2664 | * |  |  |  |  |  |  |  |  |  |
| **K020b** | *0.0091* | *-0.0059* | 0.1264 | 0.3467 | 0.2446 | * |  |  |  |  |  |  |  |  |
| **K029** | *0.0841* | *-0.0196* | 0.2806 | 0.3873 | 0.3038 | *-0.0084* | * |  |  |  |  |  |  |  |
| **T001** | 0.3094 | 0.2878 | 0.2701 | 0.3029 | 0.0459 | 0.2688 | 0.3065 | * |  |  |  |  |  |  |
| **T011** | 0.3171 | 0.3023 | 0.3012 | 0.2533 | 0.1389 | 0.2971 | 0.3295 | *0.1109* | * |  |  |  |  |  |
| **T016** | 0.4966 | 0.4906 | 0.4328 | 0.3830 | 0.1695 | 0.4136 | 0.5197 | *0.0233* | 0.1838 | * |  |  |  |  |
| **T026a** | 0.6135 | 0.6341 | 0.5610 | 0.4488 | 0.4122 | 0.5269 | 0.6698 | 0.2965 | 0.3330 | 0.2530 | * |  |  |  |
| **T027a** | 0.2832 | 0.2618 | 0.2845 | 0.2246 | 0.1503 | 0.2608 | 0.2944 | 0.1260 | *0.1046* | 0.1784 | 0.2652 | * |  |  |
| **T027b** | 0.3566 | 0.3523 | 0.3553 | 0.0969 | 0.2619 | 0.3476 | 0.3874 | 0.2688 | 0.2090 | 0.3452 | 0.4136 | 0.1825 | * |  |
| **T033a** | 0.4927 | 0.4813 | 0.4422 | 0.2063 | 0.3176 | 0.4517 | 0.5240 | 0.2898 | 0.2149 | 0.3985 | 0.4728 | *0.0105* | 0.1525 | * |
| **T033b** | 0.4135 | 0.3918 | 0.3525 | 0.2250 | 0.2885 | 0.3738 | 0.4243 | 0.2998 | 0.2710 | 0.4047 | 0.4886 | 0.1973 | 0.1905 | *0.1729* |
| **T036a** | 0.3042 | 0.2553 | 0.2778 | 0.3210 | 0.2633 | 0.2420 | 0.3247 | 0.2821 | 0.2884 | 0.4436 | 0.5728 | 0.2153 | 0.3088 | 0.3989 |
| **T040** | 0.4808 | 0.4587 | 0.4033 | 0.3061 | 0.0746 | 0.3990 | 0.4873 | *0.0070* | 0.1614 | 0.1784 | 0.5097 | 0.1918 | 0.2729 | 0.3687 |
| **T064a** | 0.3049 | 0.2996 | 0.2619 | 0.1887 | 0.1448 | 0.2823 | 0.3311 | *0.1274* | 0.1720 | 0.2458 | 0.3853 | 0.1448 | 0.1968 | 0.2121 |
| **U005** | 0.3803 | 0.3662 | 0.3804 | 0.1970 | 0.2673 | 0.3605 | 0.4037 | 0.2570 | 0.2170 | 0.3621 | 0.4347 | *0.0577* | 0.1586 | *0.0224* |
| **U012** | 0.8223 | 0.8284 | 0.7857 | 0.4063 | 0.6744 | 0.7809 | 0.8541 | 0.6477 | 0.5126 | 0.7755 | 0.8162 | 0.4135 | 0.3309 | 0.3124 |
| **U020** | 0.7997 | 0.8024 | 0.7724 | 0.4081 | 0.6772 | 0.7576 | 0.8296 | 0.6652 | 0.5489 | 0.7835 | 0.8176 | 0.4527 | 0.3411 | 0.3987 |
| **U021** | 0.8670 | 0.8826 | 0.8093 | 0.5639 | 0.6981 | 0.7861 | 0.9084 | 0.6488 | 0.5712 | 0.7879 | 0.7916 | 0.5654 | 0.5380 | 0.6803 |
| **U023a** | 0.5474 | 0.5462 | 0.5399 | 0.2139 | 0.4528 | 0.5318 | 0.5826 | 0.4532 | 0.3373 | 0.5434 | 0.5839 | 0.2268 | 0.2063 | 0.1187 |
| **U023b** | 0.5537 | 0.5527 | 0.5503 | 0.2374 | 0.4673 | 0.5399 | 0.5875 | 0.4594 | 0.3463 | 0.5515 | 0.5884 | 0.2296 | 0.2148 | 0.1320 |
| **U028** | 0.6752 | 0.6762 | 0.6628 | 0.2980 | 0.5710 | 0.6484 | 0.7085 | 0.5685 | 0.4575 | 0.6682 | 0.7022 | 0.3205 | 0.2471 | *0.1912* |
| **U030b** | 0.3059 | 0.3318 | 0.3299 | 0.1463 | 0.1854 | 0.3153 | 0.3608 | 0.1949 | 0.2070 | 0.3081 | 0.4041 | 0.1409 | 0.1438 | 0.1589 |
| **U030c** | 0.4971 | 0.4259 | 0.4093 | 0.3971 | 0.3569 | 0.3576 | 0.5205 | 0.3816 | 0.3701 | 0.6142 | 0.7523 | 0.3191 | 0.3855 | 0.5331 |
| **U037** | 0.2123 | 0.2199 | 0.2746 | 0.2151 | 0.1268 | 0.2046 | 0.2510 | 0.1737 | 0.1915 | 0.3107 | 0.4588 | *0.0822* | 0.2136 | 0.1967 |
| **U046** | 0.6696 | 0.6685 | 0.6557 | 0.3319 | 0.5701 | 0.6427 | 0.7012 | 0.5585 | 0.4535 | 0.6638 | 0.6992 | 0.3205 | 0.2797 | 0.2017 |

| **Site** | **T033b** | **T036a** | **T040** | **T064a** | **U005** | **U012** | **U020** | **U021** | **U023a** | **U023b** | **U028** | **U030b** | **U030c** | **U037** | **U046** |
| --- | --- | --- | --- | --- | --- | --- | --- | --- | --- | --- | --- | --- | --- | --- | --- |
| **T033b** | * |  |  |  |  |  |  |  |  |  |  |  |  |  |  |
| **T036a** | 0.3448 | * |  |  |  |  |  |  |  |  |  |  |  |  |  |
| **T040** | 0.3272 | 0.3657 | * |  |  |  |  |  |  |  |  |  |  |  |  |
| **T064a** | 0.2234 | 0.2470 | *0.1537* | * |  |  |  |  |  |  |  |  |  |  |  |
| **U005** | 0.1630 | 0.3084 | 0.3211 | 0.1836 | * |  |  |  |  |  |  |  |  |  |  |
| **U012** | 0.4980 | 0.7343 | 0.7412 | 0.4969 | 0.3150 | * |  |  |  |  |  |  |  |  |  |
| **U020** | 0.4871 | 0.7099 | 0.7493 | 0.5426 | 0.3515 | 0.5897 | * |  |  |  |  |  |  |  |  |
| **U021** | 0.6435 | 0.7812 | 0.8056 | 0.6016 | 0.6061 | 0.8987 | 0.8920 | * |  |  |  |  |  |  |  |
| **U023a** | 0.2772 | 0.4838 | 0.4979 | 0.3421 | 0.1038 | 0.2468 | 0.3265 | 0.6833 | * |  |  |  |  |  |  |
| **U023b** | 0.3090 | 0.4957 | 0.5146 | 0.3660 | 0.1268 | 0.2287 | 0.2958 | 0.6830 | *-0.0121* | * |  |  |  |  |  |
| **U028** | 0.3573 | 0.6051 | 0.6316 | 0.4347 | 0.1268 | 0.2951 | 0.3776 | 0.7890 | 0.0755 | 0.0717 | * |  |  |  |  |
| **U030b** | 0.1637 | 0.2897 | 0.2227 | 0.1650 | 0.1407 | 0.4372 | 0.4341 | 0.5634 | 0.2473 | 0.2610 | 0.3423 | * |  |  |  |
| **U030c** | 0.4353 | *0.1020* | 0.5291 | 0.3308 | 0.4092 | 0.8512 | 0.8274 | 0.9212 | 0.5814 | 0.5894 | 0.7094 | 0.3857 | * |  |  |
| **U037** | 0.2233 | 0.1863 | 0.2369 | 0.1579 | 0.1464 | 0.5910 | 0.5727 | 0.6865 | 0.3384 | 0.3564 | 0.4496 | 0.1225 | 0.2518 | * |  |
| **U046** | 0.3738 | 0.5980 | 0.6259 | 0.4460 | 0.1230 | 0.3307 | 0.4025 | 0.7849 | 0.0877 | 0.0660 | *0.0585* | 0.3418 | 0.6993 | 0.4397 | * |
