# Supplementary material for: The population genetic structure of Biomphalaria choanomphala in Lake Victoria, East Africa: implications for schistosomiasis transmission
Source: Parasit Vectors. 2014 Nov 19;7:524. doi: 10.1186/s13071-014-0524-4 (PMC4254209; doi:10.1186/s13071-014-0524-4)
Supplement: Additional file 8: Table S4. — Full table of pairwise FST values per site for 16S marker. Non-significant pairwise distances are given in italics. [file 13071_2014_524_MOESM8_ESM.doc]

| **Site** | **K001a** | **K002a** | **K006a** | **K006b** | **K013b** | **K020b** | **K029** | **T001** | **T011** | **T016** | **T026a** | **T027a** | **T027b** | **T033a** | **T033b** |
| --- | --- | --- | --- | --- | --- | --- | --- | --- | --- | --- | --- | --- | --- | --- | --- |
| **K001a** | * |  |  |  |  |  |  |  |  |  |  |  |  |  |  |
| **K002a** | *-0.0183* | * |  |  |  |  |  |  |  |  |  |  |  |  |  |
| **K006a** | 0.2198 | 0.1433 | * |  |  |  |  |  |  |  |  |  |  |  |  |
| **K006b** | 0.4721 | 0.4043 | 0.4175 | * |  |  |  |  |  |  |  |  |  |  |  |
| **K013b** | 0.4194 | 0.3146 | 0.2726 | 0.3387 | * |  |  |  |  |  |  |  |  |  |  |
| **K020b** | *0.0072* | *0.0035* | *0.1652* | 0.4413 | 0.3287 | * |  |  |  |  |  |  |  |  |  |
| **K029** | *0.0518* | *0.0311* | 0.3598 | 0.4479 | 0.4249 | *-0.0220* | * |  |  |  |  |  |  |  |  |
| **T001** | 0.3746 | 0.2674 | 0.2267 | 0.2440 | *0.0276* | 0.2866 | 0.3719 | * |  |  |  |  |  |  |  |
| **T011** | 0.4911 | 0.3889 | 0.4012 | 0.2604 | 0.1988 | 0.4282 | 0.4830 | 0.1328 | * |  |  |  |  |  |  |
| **T016** | 0.5350 | 0.4018 | 0.3825 | 0.3724 | 0.1532 | 0.4525 | 0.5573 | *0.0516* | 0.2912 | * |  |  |  |  |  |
| **T026a** | 0.5231 | 0.3589 | 0.3159 | 0.4041 | 0.1861 | 0.4457 | 0.5827 | 0.1102 | 0.3468 | *0.1731* | * |  |  |  |  |
| **T027a** | 0.3487 | 0.2397 | 0.2590 | 0.2057 | 0.1752 | 0.2975 | 0.3516 | 0.0999 | 0.2085 | 0.2179 | 0.1910 | * |  |  |  |
| **T027b** | 0.5354 | 0.4651 | 0.4914 | 0.1177 | 0.4271 | 0.5062 | 0.5123 | 0.3289 | 0.3462 | 0.4618 | 0.4950 | 0.2680 | * |  |  |
| **T033a** | 0.5550 | 0.4546 | 0.5328 | 0.2339 | 0.4285 | 0.5228 | 0.5477 | 0.3366 | 0.3893 | 0.5020 | 0.5594 | 0.1513 | 0.2232 | * |  |
| **T033b** | 0.4200 | 0.3173 | 0.3177 | 0.1965 | 0.1529 | 0.3506 | 0.4019 | *0.0509* | 0.1641 | 0.1969 | 0.2315 | *0.0538* | 0.2641 | 0.2178 | * |
| **T036a** | 0.2901 | 0.1851 | 0.1800 | 0.3316 | 0.2149 | 0.2768 | 0.3466 | 0.1676 | 0.2880 | 0.2434 | 0.1974 | 0.1295 | 0.4038 | 0.3649 | 0.2460 |
| **T040** | 0.4392 | 0.3192 | 0.2808 | 0.3071 | *-0.0018* | 0.3479 | 0.4548 | *-0.0089* | 0.1766 | 0.1264 | 0.1506 | 0.1525 | 0.3969 | 0.4254 | 0.1339 |
| **T064a** | 0.3797 | 0.2815 | 0.2743 | 0.1885 | 0.1499 | 0.3088 | 0.3552 | *0.0880* | 0.2006 | 0.2203 | 0.2210 | *0.0803* | 0.2804 | 0.2912 | 0.0890 |
| **U005** | 0.4759 | 0.3981 | 0.4325 | 0.1579 | 0.3418 | 0.4358 | 0.4502 | 0.2304 | 0.3215 | 0.3618 | 0.4276 | 0.1685 | 0.2117 | 0.1268 | 0.1270 |
| **U012** | 0.6561 | 0.5846 | 0.6315 | 0.2907 | 0.5648 | 0.6312 | 0.6395 | 0.4692 | 0.5248 | 0.5606 | 0.6373 | 0.3755 | 0.3128 | 0.3309 | 0.3782 |
| **U020** | 0.8142 | 0.7279 | 0.8067 | 0.3966 | 0.7192 | 0.7985 | 0.8220 | 0.6391 | 0.6788 | 0.7567 | 0.7858 | 0.5009 | 0.4281 | 0.5578 | 0.5222 |
| **U021** | 0.7162 | 0.5198 | 0.6317 | 0.4483 | 0.3989 | 0.6665 | 0.7701 | 0.3088 | 0.4718 | 0.4103 | 0.2518 | 0.2819 | 0.5544 | 0.6686 | 0.3469 |
| **U023a** | 0.5026 | 0.4360 | 0.4673 | 0.1126 | 0.4063 | 0.4745 | 0.4770 | 0.3149 | 0.3382 | 0.4256 | 0.4675 | 0.2074 | 0.1451 | 0.1445 | 0.2116 |
| **U023b** | 0.5345 | 0.4670 | 0.5127 | 0.1791 | 0.4475 | 0.5112 | 0.5104 | 0.3700 | 0.4016 | 0.4883 | 0.5285 | 0.2330 | 0.2143 | 0.1141 | 0.2629 |
| **U028** | 0.5667 | 0.4852 | 0.5359 | 0.1251 | 0.4430 | 0.5342 | 0.5418 | 0.3569 | 0.3792 | 0.4973 | 0.5331 | 0.2156 | 0.1322 | 0.1315 | 0.2228 |
| **U030b** | 0.4162 | 0.3574 | 0.3597 | 0.1892 | 0.2362 | 0.3866 | 0.4100 | 0.2032 | 0.2502 | 0.3447 | 0.3740 | 0.2301 | 0.2153 | 0.2663 | 0.2161 |
| **U030c** | 0.3806 | 0.2598 | 0.2791 | 0.3129 | 0.1593 | 0.2899 | 0.3671 | 0.1007 | 0.2302 | 0.2320 | 0.2605 | 0.1378 | 0.3930 | 0.3873 | 0.1825 |
| **U037** | 0.3870 | 0.3068 | 0.3181 | 0.1906 | 0.1626 | 0.3314 | 0.3842 | 0.1104 | 0.1633 | 0.2767 | 0.3218 | 0.1140 | 0.2416 | 0.2129 | 0.1373 |
| **U046** | 0.5258 | 0.4489 | 0.4991 | 0.1482 | 0.4387 | 0.5054 | 0.5068 | 0.3509 | 0.3907 | 0.4802 | 0.5087 | 0.2287 | 0.1643 | 0.1688 | 0.2622 |

| **Site** | **T036a** | **T040** | **T064a** | **U005** | **U012** | **U020** | **U021** | **U023a** | **U023b** | **U028** | **U030b** | **U030c** | **U037** | **U046** |
| --- | --- | --- | --- | --- | --- | --- | --- | --- | --- | --- | --- | --- | --- | --- |
| **T036a** | * |  |  |  |  |  |  |  |  |  |  |  |  |  |
| **T040** | 0.1866 | * |  |  |  |  |  |  |  |  |  |  |  |  |
| **T064a** | 0.2203 | *0.1378* | * |  |  |  |  |  |  |  |  |  |  |  |
| **U005** | 0.3360 | 0.3304 | 0.2112 | * |  |  |  |  |  |  |  |  |  |  |
| **U012** | 0.5108 | 0.5550 | 0.4094 | 0.1908 | * |  |  |  |  |  |  |  |  |  |
| **U020** | 0.6356 | 0.7269 | 0.5408 | 0.3961 | 0.4976 | * |  |  |  |  |  |  |  |  |
| **U021** | 0.3541 | 0.3946 | 0.3153 | 0.5171 | 0.6976 | 0.8451 | * |  |  |  |  |  |  |  |
| **U023a** | 0.3713 | 0.3884 | 0.2451 | 0.0963 | 0.1881 | 0.3173 | 0.5249 | * |  |  |  |  |  |  |
| **U023b** | 0.4097 | 0.4394 | 0.2905 | *0.0581* | 0.2115 | 0.3901 | 0.5873 | *0.0385* | * |  |  |  |  |  |
| **U028** | 0.4262 | 0.4346 | 0.2671 | 0.0638 | 0.2482 | 0.3600 | 0.6018 | *0.0400* | *0.0191* | * |  |  |  |  |
| **U030b** | 0.2765 | 0.1953 | 0.2125 | 0.2274 | 0.4021 | 0.5452 | 0.4809 | 0.2641 | 0.2818 | 0.2686 | * |  |  |  |
| **U030c** | 0.0828 | 0.1461 | 0.1727 | 0.3175 | 0.5302 | 0.6820 | 0.4586 | 0.3576 | 0.4090 | 0.4117 | 0.2808 | * |  |  |
| **U037** | 0.2051 | 0.1055 | 0.1651 | 0.1840 | 0.4108 | 0.5763 | 0.4881 | 0.2292 | 0.2575 | 0.2301 | *0.0795* | 0.1594 | * |  |
| **U046** | 0.3836 | 0.4213 | 0.2642 | 0.0709 | 0.2173 | 0.2689 | 0.5701 | *0.0500* | *0.0360* | *0.0229* | 0.2472 | 0.4044 | 0.2273 | * |
