# Supplementary material for: The population genetic structure of Biomphalaria choanomphala in Lake Victoria, East Africa: implications for schistosomiasis transmission
Source: Parasit Vectors. 2014 Nov 19;7:524. doi: 10.1186/s13071-014-0524-4 (PMC4254209; doi:10.1186/s13071-014-0524-4)
Supplement: Additional file 9: Table S5. — Full table of pairwise FST values per site for microsatellite markers. Non-significant pairwise distances are given in italics. [file 13071_2014_524_MOESM9_ESM.doc]

| **Site** | **K001a** | **K002a** | **K006a** | **K006b** | **K013b** | **K020b** | **T001** | **T011** | **T016** | **T026a** | **T027a** | **T027b** |
| --- | --- | --- | --- | --- | --- | --- | --- | --- | --- | --- | --- | --- |
| **K001a** | * |  |  |  |  |  |  |  |  |  |  |  |
| **K002a** | *0.0277* | * |  |  |  |  |  |  |  |  |  |  |
| **K006a** | *0.0167* | *-0.0165* | * |  |  |  |  |  |  |  |  |  |
| **K006b** | 0.1266 | *0.0454* | *0.1086* | * |  |  |  |  |  |  |  |  |
| **K013b** | 0.1235 | 0.1002 | *0.0898* | 0.1735 | * |  |  |  |  |  |  |  |
| **K020b** | 0.0600 | 0.0420 | *0.0098* | 0.1155 | 0.1594 | * |  |  |  |  |  |  |
| **T001** | *0.0729* | *0.0755* | *0.0783* | 0.1828 | *0.1750* | 0.1377 | * |  |  |  |  |  |
| **T011** | 0.0734 | 0.1043 | *0.1619* | 0.2034 | 0.1939 | 0.1255 | *0.0851* | * |  |  |  |  |
| **T016** | 0.0876 | 0.0810 | *0.0894* | 0.1967 | 0.1338 | 0.1477 | *0.0406* | 0.1316 | * |  |  |  |
| **T026a** | 0.0921 | 0.1307 | *0.2258* | 0.2226 | 0.2321 | 0.1638 | *0.1346* | *0.1695* | 0.1197 | * |  |  |
| **T027a** | 0.0594 | 0.0500 | *0.0245* | 0.0900 | 0.1069 | 0.0987 | 0.1019 | 0.1788 | 0.0912 | 0.1350 | * |  |
| **T027b** | 0.1245 | 0.1323 | *0.1425* | 0.0948 | 0.1875 | 0.1641 | 0.1962 | 0.2031 | 0.2038 | 0.2552 | 0.1080 | * |
| **T033a** | 0.0782 | 0.0511 | *0.0859* | 0.0848 | 0.1560 | 0.0758 | *0.1416* | 0.2097 | 0.1554 | 0.1921 | 0.0753 | 0.1162 |
| **T033b** | 0.1242 | 0.1373 | *0.1126* | *0.0785* | 0.1892 | 0.1502 | *0.1451* | 0.1466 | 0.1817 | 0.2355 | 0.1182 | *0.0214* |
| **T036a** | 0.1090 | 0.0837 | *0.1379* | 0.1551 | 0.1186 | 0.1310 | 0.1726 | 0.1239 | 0.1546 | 0.1833 | 0.1353 | 0.1061 |
| **T040** | 0.1033 | 0.0571 | *-0.0088* | *0.0887* | 0.1151 | 0.1281 | 0.1131 | 0.2041 | 0.0910 | 0.1594 | *0.0099* | 0.1081 |
| **T064a** | 0.0594 | 0.0381 | *0.0978* | 0.1286 | 0.1370 | 0.0699 | *0.0994* | *0.0399* | 0.1096 | 0.1771 | 0.1322 | 0.1529 |
| **U005** | 0.0471 | 0.0432 | *0.0565* | 0.1519 | 0.1413 | 0.0950 | *0.0875* | 0.0540 | 0.0836 | 0.0801 | 0.1059 | 0.1711 |
| **U020** | *0.0441* | 0.1082 | *0.1841* | 0.1529 | 0.1651 | 0.1385 | 0.1394 | 0.1488 | 0.1569 | 0.1195 | 0.0946 | 0.1940 |
| **U021** | *0.0365* | 0.1119 | *0.1269* | 0.1626 | 0.1615 | 0.1366 | 0.1434 | 0.1483 | 0.1823 | 0.1874 | 0.1241 | 0.1687 |
| **U023a** | 0.0869 | 0.0790 | *0.0130* | *0.0688* | 0.0788 | 0.1245 | 0.1466 | 0.1897 | 0.1656 | 0.2171 | 0.0776 | 0.0782 |
| **U023b** | *0.0401* | 0.0607 | *-0.0218* | *0.0596* | 0.1135 | 0.0931 | *0.1180* | 0.1550 | 0.1280 | 0.1520 | 0.0503 | 0.0433 |
| **U028** | 0.0440 | 0.0326 | *0.0192* | 0.0675 | 0.0959 | 0.0749 | *0.0835* | 0.1095 | 0.0821 | 0.1411 | 0.0495 | 0.0802 |
| **U030b** | *0.0918* | 0.0586 | *-0.0182* | *0.0720* | *0.1891* | 0.0800 | *0.1778* | *0.1818* | 0.1571 | 0.2453 | 0.1064 | 0.1123 |
| **U037** | 0.0432 | 0.0194 | *0.0485* | 0.1137 | 0.1213 | 0.0454 | *0.0685* | 0.0912 | 0.1135 | 0.1618 | 0.0962 | 0.1533 |
| **U046** | 0.0378 | 0.0182 | *0.0594* | 0.1228 | 0.1322 | 0.0817 | 0.1055 | 0.0718 | 0.0989 | 0.1305 | 0.1001 | 0.1364 |

| **Site** | **T033a** | **T033b** | **T036a** | **T040** | **T064a** | **U005** | **U020** | **U021** | **U023a** | **U023b** | **U028** | **U030b** | **U037** | **U046** |
| --- | --- | --- | --- | --- | --- | --- | --- | --- | --- | --- | --- | --- | --- | --- |
| **T033a** | * |  |  |  |  |  |  |  |  |  |  |  |  |  |
| **T033b** | 0.1439 | * |  |  |  |  |  |  |  |  |  |  |  |  |
| **T036a** | 0.1331 | 0.1563 | * |  |  |  |  |  |  |  |  |  |  |  |
| **T040** | 0.0755 | 0.1166 | 0.1335 | * |  |  |  |  |  |  |  |  |  |  |
| **T064a** | 0.1035 | 0.1165 | 0.0959 | 0.1123 | * |  |  |  |  |  |  |  |  |  |
| **U005** | 0.0840 | 0.1430 | 0.0921 | 0.0959 | 0.0466 | * |  |  |  |  |  |  |  |  |
| **U020** | 0.1493 | 0.1850 | 0.1665 | 0.1479 | 0.1106 | 0.1358 | * |  |  |  |  |  |  |  |
| **U021** | 0.1598 | 0.1550 | 0.1765 | 0.1562 | 0.1223 | 0.1311 | 0.0922 | * |  |  |  |  |  |  |
| **U023a** | 0.1010 | *0.0264* | 0.1452 | 0.0695 | 0.0830 | 0.1229 | 0.1444 | 0.1267 | * |  |  |  |  |  |
| **U023b** | *0.0598* | *0.0339* | 0.1007 | *0.0472* | 0.1004 | 0.0856 | 0.1234 | 0.0988 | *-0.0038* | * |  |  |  |  |
| **U028** | *0.0387* | 0.0575 | 0.0770 | 0.0555 | 0.0587 | 0.0405 | 0.1370 | 0.1246 | *0.0364* | *0.0173* | * |  |  |  |
| **U030b** | *0.0925* | *0.0732* | 0.1622 | 0.1092 | *0.0528* | *0.0901* | *0.1616* | 0.1425 | *0.0234* | *-0.0004* | 0.0709 | * |  |  |
| **U037** | 0.0565 | 0.1218 | 0.1092 | 0.1107 | *0.0185* | 0.0380 | 0.1431 | 0.1189 | 0.0743 | 0.0625 | *0.0220* | *0.0548* | * |  |
| **U046** | 0.0829 | 0.1206 | 0.0989 | 0.1031 | *0.0143* | *0.0326* | 0.1344 | 0.1252 | 0.0654 | 0.0605 | 0.0489 | *0.0615* | *0.0145* | * |
